# Toxicity and Transcriptome Sequencing (RNA-seq) Analyses of Adult Zebrafish in Response to Exposure Carboxymethyl Cellulose Stabilized Iron Sulfide Nanoparticles

**DOI:** 10.1038/s41598-018-26499-x

**Published:** 2018-05-24

**Authors:** Min Zheng, Jianguo Lu, Dongye Zhao

**Affiliations:** 1 Environmental Engineering Program, Department of Civil Engineering, Auburn University, Auburn, AL 36849 USA; 2 School of Marine Sciences, Sun Yat-sen University, Guangdong, 510275 China; 3 Beijing University of Civil Engineering and Architecture, Beijing, 100044 PR China

## Abstract

Increasing utilization of stabilized iron sulfides (FeS) nanoparticles implies an elevated release of the materials into the environment. To understand potential impacts and underlying mechanisms of nanoparticle-induced stress, we used the transcriptome sequencing (RNA-seq) technique to characterize the transcriptomes from adult zebrafish exposed to 10 mg/L carboxymethyl cellulose (CMC) stabilized FeS nanoparticles for 96 h, demonstrating striking differences in the gene expression profiles in liver. The exposure caused significant expression alterations in genes related to immune and inflammatory responses, detoxification, oxidative stress and DNA damage/repair. The complement and coagulation cascades Kyoto encyclopedia of genes and genomes (KEGG) pathway was found significantly up-regulated under nanoparticle exposure. The quantitative real-time polymerase chain reaction using twelve genes confirmed the RNA-seq results. We identified several candidate genes commonly regulated in liver, which may serve as gene indicators when exposed to the nanoparticles. Hepatic inflammation was further confirmed by histological observation of pyknotic nuclei, and vacuole formation upon exposure. Tissue accumulation tests showed a 2.2 times higher iron concentration in the fish tissue upon exposure. This study provides preliminary mechanistic insights into potential toxic effects of organic matter stabilized FeS nanoparticles, which will improve our understanding of the genotoxicity caused by stabilized nanoparticles.

## Introduction

Iron sulfide (FeS) nanoparticles (NPs) have attracted increasing attention in the environmental remediation field due to their high adsorption capacity and reduction power for a variety of important pollutants^1^. It has been used for removal or immobilization of a broad spectrum of pollutants (e.g., heavy metals, metalloids, oxyanions, radionuclides, chlorinated organic compounds, nitroaromatic compounds, and polychlorinated biphenyls) in soil and water^2–11^.

To facilitate *in situ* remediation of contaminated soil and groundwater, stabilized NPs are often employed. Typically, stabilized NPs are prepared by coating certain organic molecules on NPs to prevent the NPs from aggregation. Of various stabilizers reported so far, carboxymethyl cellulose (CMC) represents one of the best stabilizers, which is not only effective, but also green and inexpensive. For instance, Gong *et al*.^12,13^ prepared and tested CMC-stabilized FeS NPs for highly effective removal/immobilization of mercury in soil and groundwater. In addition to purposely stabilized NPs, NPs in the environment may become “passively” stabilized by dissolved organic matter (DOM) in the natural environmental systems^14–16^. Compared to the non-stabilized counterparts, stabilized NPs are often much smaller in size, more mobile in the environment, and more reactive^17^. They are more transportable in soil or water^18,19^, and may pose broader and more severe toxic effects on the environment and biota. Yet, little information is available on the toxicity of stabilized NPs.

To assure environmentally safe application of stabilized FeS NPs, it is important to understand the potential environmental risks to the ecosystem and human health. The aquatic environment is particularly vulnerable to manufactured NPs, as it acts as a sink for virtually all environmental contaminants^20^. Consequently, understanding the fate and eco-toxicological risks of NPs in the aquatic systems is urgently needed.

However, our knowledge about the toxicity of FeS NPs, especially, stabilized FeS NPs, is very limited. Bare FeS NPs were reported to bind with DNA, limiting the ability of DNA to interact with other nucleic acids and amino acids^21^. Furthermore, it was shown that FeS NPs at concentrations above its solubility limit may pose genotoxicity by reacting with polynucleic acids, and may nick DNA molecules at concentrations below its solubility limit^22^. It was also reported that FeS particles can suppress the growth of microorganisms and plants. For instance, in the presence of 2 × 10^−5^ M to 5 × 10^−3^ M of non-stabilized FeS NPs, *E. coli* growth rate was reduced under anaerobic conditions^23^. FeS particles may impede nutrients uptake and were found partially responsible for reduced seed production and viability when precipitated on the roots of wild rice plants^24^. However, the underlying molecular mechanisms governing the genotoxicity of FeS or its NPs remain largely unknown.

The recently developed transcriptome sequencing (RNA-seq) technique provides a powerful tool for investigating into the genotoxic effects and the molecular mechanisms in organisms after a chemical exposure. It is particularly useful for studying emerging environmental pollutants with limited toxicological information, such as NPs, since it allows for a global examination of biological response through gene expression. Zebrafish (*Danio rerio*), whose genome has been completely sequenced, is a common model organism for investigating genotoxic effects of chemicals, and it has been used in studying the eco-toxicological effects of engineered NPs^25,26^.

As the environmental applications of stabilized NPs continue to rise, it becomes critical to understand their potential environmental implications. To this end, this study aimed to investigate the stress response of adult zebrafish to CMC-stabilized FeS NPs (CMC-FeS) through the state of the art RNA-seq technique together with the tissue burdens and histological alternation assessments. Differentially expressed genes (DEGs) profiles, gene ontology (GO) and Kyoto encyclopedia of genes and genomes (KEGG) pathways were acquired and analyzed to ascertain genomic responses to the specific stress under CMC-FeS exposure. The reliability of the transcriptomic results were validated by RT-qPCR of selected genes.

## Materials and Methods

### Chemicals

Iron sulfate heptahydrate (FeSO_4_⋅7H_2_O) and CMC (MW = 90 000 in the sodium form, degree of substitution = 0.7) were purchased from Acros Organics (Morris Plains, NJ, USA). Sodium sulfide nonahydrate (Na_2_S·9H_2_O), hydrogen peroxide (H_2_O_2_), acetic acid, formalin and xylene were obtained from Fisher Scientific (Fair Lawn, NJ, USA). Nitric acid was obtained from Mallinckrodt Chemical (St. Louis, MO, USA). All chemicals are of the ACS reagent grade. All solutions were prepared with local tap water. To avoid effect of chlorine, the tap water was first dechlorinated under aeration at pH 6.0 for ~7 days. Table S1 in Supplementary Information (SI) gives the relevant water quality parameter.

### Synthesis and characterization of CMC-FeS nanoparticles

Stabilized CMC-FeS NPs were prepared in 1000 mL flask with nitrogen purging/mixing. First, a CMC solution (0.1%, w/w) was prepared by dissolving CMC with tap water and the solution was purged with purified N_2_ (>99%) for half an hour to remove dissolved oxygen (DO). Likewise, a solution of 0.0114 M FeSO_4_ and 0.0152 M Na_2_S were also prepared with N_2_ purged tap water. Then, the FeSO_4_ solution was mixed with the CMC solution to yield a desired concentration of iron and the stabilizer. In this work, 0.001% (w/w) of the CMC was used to stabilize 10 mg/L FeS nanoparticles (i.e. a CMC-to-FeS molar ratio of 0.0005). The mixture was then purged with purified N_2_ for half an hour to complete the formation of Fe^2+^-CMC complexes. Then, the Na_2_S solution was introduced into Fe^2+^-CMC solution at an Fe-to-S molar ratio of 1:1 to yield the FeS nanoparticles. The nanoparticles were characterized within 1 h of preparation.

The morphology of CMC-FeS was determined by Transmission Electron Microscopy (TEM) at 200 KV accelerating voltage (JEM-2100, JEOL, Tokyo, Japan). The hydrodynamic diameter and zeta potential (ζ) were determined by dynamic light scattering (DLS) using a Malvern Zetasizer Nano ZS (Malvern Instruments, Worcestershire, U.K.). In this paper, all data on particle sizes are given as mean ± standard deviation.

### Zebrafish experimental study

All procedures involving the handling and treatment of fish used during this study were approved by the Animal Care and Use Committee at the Heilongjiang River Fisheries Research Institute (ACUC-HRFRI). All experiments were conducted in accordance with the relevant guidelines and regulations.

The zebrafish (*Danio rerio*) were obtained from the Heilongjiang River Fisheries Research Institute Zebrafish Facilities. Adult zebrafish of both sexes with an average age of 6 months, average weight of 0.61 ± 0.10 g and average length of 44.27 ± 2.77 mm were selected for the study. Before the tests, the fish were fed daily with commercially purchased fish food and maintained in aquaria at a temperature of 25 ± 1 °C with a 14 h:10 h light and dark cycle for four weeks. The fish were removed from aquaria and placed in static tanks and fasted for 24 h prior to each experiment, and no fish died before the intended exposure.

The sub-lethal concentrations (LC_50_) were determined following the Organization for Economic Cooperation and Development (OECD) guidelines for testing chemicals^27^. Five different concentrations of CMC-FeS suspensions (100, 50, 25, 10 and 5 mg/L as total Fe) were prepared prior to use. The zebrafish were randomly assigned to 5 groups (10 fish each) and in turn exposed to an assigned concentration for 96 h in 1 L beaker containing 1 L of a test solution. The sixth group of 10 fish was used as no-dose control. Each treatment was run in triplicates under the same conditions with the natural light/dark cycle. For maintaining the quality of water, the fish were not fed for 24 h prior to the experiments and during the experiments. The water temperature was maintained at 25 ± 1 °C and pH in the range of 6.8–7.3. The number of dead fish was recorded every 12 h and they were removed from the treatment beaker immediately to avoid contamination.

After the LC_50_ was determined, approximately half of the LC_50_ dose, i.e., the LC_25_ (LC_25_ = 10.5 mg/L as total Fe) was applied to 10 fresh zebrafish in 1 L of a test solution/suspension for 96 h. In order to maintain the constant concentration of the nanoparticles, the test solutions/suspensions were replaced every 24 hours^28^, where the fish were carefully removed from the solution/suspensions with a fish net without any further clean of the adhered NPs in order to avoid unnecessary perturbance of fish by handling, and then added a batch of freshly prepared solution/suspensions. The variation of total iron of FeS NPs were quantified by ICP-MS to be less than 0.2% at time 0 and 24 h in the system, and thus, residual sorbed NPs on fish did not pose a significant effect on the exposure concentration. All treatments were carried out in triplicate and no mortality was observed during the exposure experiments. After the 96 h of exposure, zebrafish in each treatment were rinsed with tap water three times to remove the nanoparticles on fish surface for further testing. To measure the amount of Fe^2+^ released from CMC-FeS into the solution, control testing suspensions were filtered at time zero and 24 h of preparation using a 25 nm Millipore membrane filter (Millipore Corp., Billerica, MA, USA) and dissolved Fe concentrations were analyzed using an Agilent 7500 cx ICP-MS (Agilent Technologies, USA) equipped with an Octopole Reaction System (ORS).

### Tissue accumulations

After the 96 h of exposure, three zebrafish were removed from each of the triplicate testing beakers including the control, and then euthanized with tricaine methanesulfonate (MS222). The tissue samples were weighed (0.41 ± 0.10 g), and then digested for analyzing the Fe content. The digestion was performed in a MarXpress microwave system (CEM, USA). A homogenized tissue sample was transferred into a PTFE (polytetrafluoroethylene) vessel. Then, 5 mL HNO_3_, 2 mL H_2_O_2_ and 3 mL ultra-pure water were added, and then the temperature was ramped to 185 °C in 10.5 min, and kept at this temperature for 14.5 min. After cooling to room temperature, the samples were filtered using a 0.45 µm filter. Finally, 0.5 mL of a 100 mg /L internal standard Mix solution (Agilent Technologies, USA) was added into the filtrates and then diluted to a final volume of 50 mL using ultra-pure water. The samples were then analyzed for total Fe concentration via an ICP-MS system. Fish tissues in the control were also digested in the same way.

### Histopathology of liver tissue

Two randomly selected fish were removed from each of the triplicate treatment beakers at 96 h of exposure. Fish were euthanized in buffered MS222 and subsequently the liver tissues were dissected. The tissues were fixed in the Davidson’s Fixative (95% Ethanol, Acetic acid, formalin and deionized water) in cassettes for 48 hours. Then the tissues were stored in 70% ethanol. Further, the tissues were dehydrated using graded ethanol series 80%, 90%, 95% and 100% ethanol for 60 mins each, then another 30 mins in 100% ethanol, followed by 60 mins in Xylene, and another 60 mins in fresh Xylene. The carcasses were subsequently transferred to a Tissue Embedding System, and the tissues were embedded in paraffin. The tissue blocks were sectioned into 6 μm thick ribbons with microtome. The ribbons were transferred to a water bath set at 45 °C. Selected tissue sections were placed on slides, which were set vertical to air dry and then placed on a slide warmer set at 45 °C until completely dry. The slides were then stained using the hematoxylin and eosin (H&E) stain by the method of Shehand and Hrapchak^29^. The stained slides were mounted and covered with a coverslip. Lastly, the slides were observed using a light microscopy on an Olympus BX40 microscope, and photomicrographs were taken using an Olympus BX53 digital camera.

### High-throughput transcriptomic sequencing

#### Total RNA isolation and illumina sequencing

Total RNAs were isolated from triplicates of liver tissues (each replicate consisted of tissues pooled from three fishes) at control and treated zebrafish groups. The tissues were homogenized in the TRIzol® Reagent and total RNA was extracted using RNeasy Mini Kit (Qiagen, Hilden, Germany) following the manufacturer’s protocol. The quantity and quality of RNA were examined by Thermo ScientificTM NanoDropTM 8000 Spectrophotometer and Agilent 2100 Bioanalyzer (Agilent Technologies, Santa Clara, CA, U.S.). Only RNA with OD 260/280 ≥ 1.8 and RNA integrity number ≥ 7 were selected for the subsequent experiments. Equal quantities of high quality RNA from each tissue sample were pooled together for cDNA synthesis and sequencing.

After generating the clusters, library sequencing was performed on an Illumina Hiseq. 4000 platform, to create paired-end reads with a length of 150 bp.

#### Bioinformatics analyses

The quality control of RNA-Seq data was conducted using the NGS QC Toolkit^30^ with default parameters. Clean paired-end reads were aligned to the zebrafish reference genome sequence, GRCz10 version^31^, using TopHat^32^.

To identify differential expression genes (DEGs) between two different samples, the expression level for each transcript was calculated using the fragments per kilobase of exon according to the million mapped reads (FRKM) method. Cuffdiff (http://cufflinks.cbcb.umd.edu/)^33^ was used for the differential expression analysis. The DEGs between two samples were selected based on the following criteria: 1) the fold change was greater than 2, and 2) the false discovery rate (FDR) was less than 0.05. To understand the functions of the differentially expressed genes, gene ontology (GO) functional enrichment and Kyoto encyclopedia of genes and genomes (KEGG) pathway analysis were carried out by Goatools (https://github.com/tanghaibao/Goatools) and KOBAS (http://kobas.cbi.pku.edu.cn/home.do)^34^. DEGs were significantly enriched in the GO terms and metabolic pathways when their Bonferroni-corrected P-value was less than 0.05.

### Experimental validation by qRT-PCR

In order to validate the expression pattern of DEGs identified by RNA-Seq, we selected twelve genes from DEGs potentially associated with immune and inflammatory response, detoxification, oxidative stress, and DNA damage/repair for qPCR validation, including *flot2a*, *cp*, *stat2*, *tsc22d3*, *sgk1*, *sod3a*, *cyp1a*, *abcb4*, *krt18*, *pdia4*, *rad51b*, and *orc1*. Total RNA was extracted using the TRIzol reagent following the manufacturer’s instructions. The RNA quality was assessed using 1% agarose electrophoresis and by measuring the 260/280 nm absorbance ratios. After purification using DNase I (Promega) to remove genomic DNA contamination, the total RNA was reverse-transcribed into cDNA and the gene transcription levels were analyzed using a SYBR Green PCR kit (Toyobo, Osaka, Japan) on an ABI PRISM 7300 Sequence Detector system (Perkin-Elmer, Applied Biosystems). Primer sequences of the selected genes were designed using Primer 3 software (http://frodo.wi.mit.edu/) (Supplementary Information (SI) Table S2). The relative gene transcription levels were calculated using the 2^−ΔΔCT^ method; beta-actin was used as the reference gene and its transcription was constant among exposure groups. The Pearson’s correlation of log_10_ (fold-change) between qPCR and RNA-Seq was 0.80, indicating the accuracy and reliability of the RNA-Seq based transcriptome analysis.

### Statistical Analysis

The statistical analysis approach described by Audic and Claverie^35^ was performed to compare the difference in gene expression. The false discovery rate (FDR) was used to determine the threshold P-values in multiple testing and analysis^35^. A FDR <0.05 and absolute value of the log 2 (fold change) >1 were used as the threshold to determine the statistical significant difference in gene expression^36^. For GO enrichment analysis, the Bonferroni correction was performed, and the corrected P-value (<0.05) was used to determine the significant enrichment of the gene sets. For KEGG enrichment analysis, a FDR <0.05 was used as the threshold to judge the significant enrichment of the gene sets^35^. The Student *t* test was carried out to determine whether there was a significant difference between experimental variables. A P-value < 0.05 was considered statistically significant.

## Results

### Characterization of CMC-FeS

**Figure 1:**
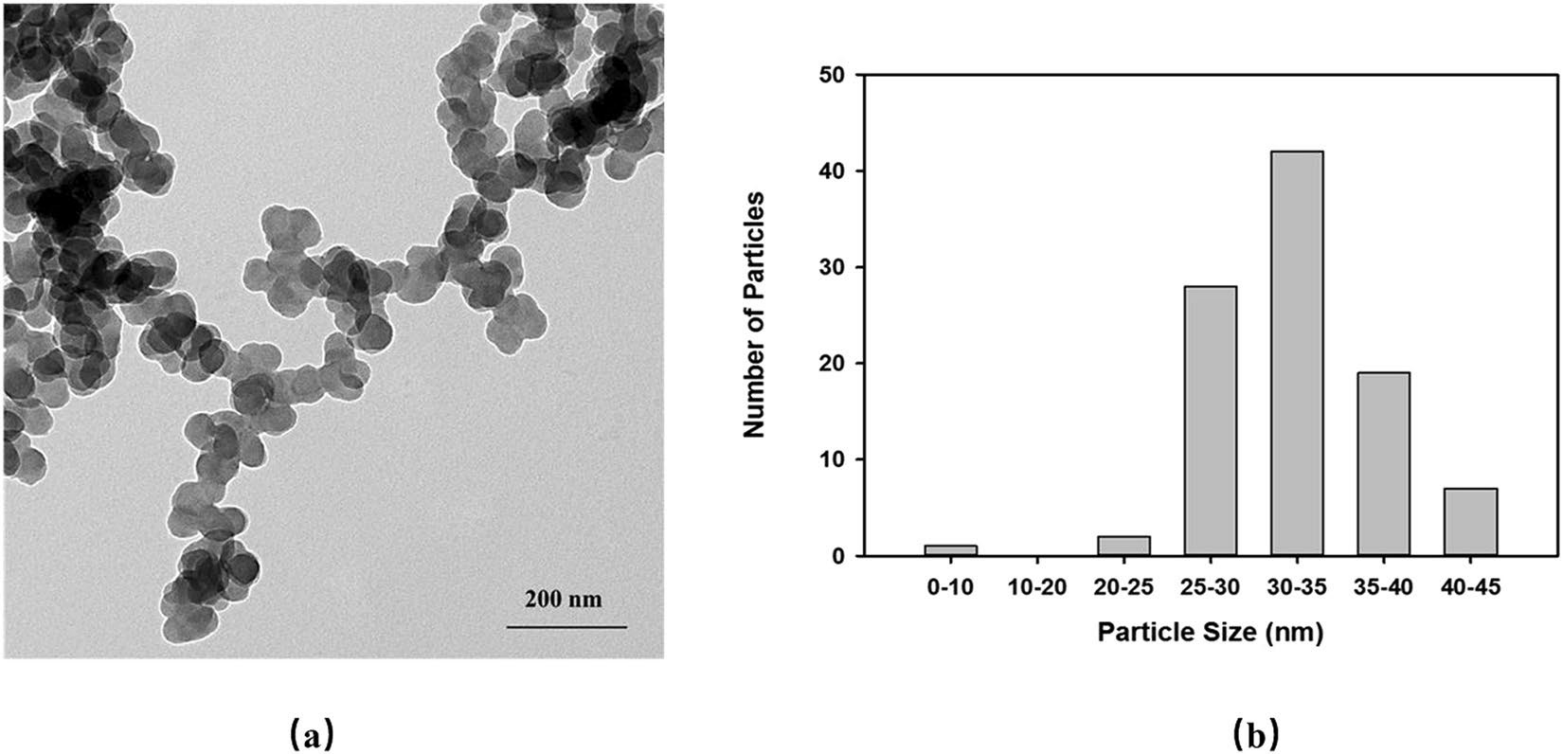
Transmission electron microscopy (TEM) image (**a**) and the histogram of size distribution (**b**) of CMC-stabilized FeS nanoparticles (FeS = 10 mg/L, CMC = 0.001 wt.%). The mean particle size is 32.18 ± 5.25 nm.

Transmission electron microscopy (TEM) was used to confirm size of synthetic CMC-FeS. As shown in Fig. 1a, the nanoparticles appear mainly spherical. Figure 1b shows the size distribution of CMC-FeS, estimated with the ImageJ software. The mean particle size was 32.2 ± 5.3 nm. The mean hydrodynamic diameter of was 133.5 ± 0.1 nm as measured by DLS. The zeta-potential of CMC-FeS was −37.95 ± 0.35 mV, indicating the stable dispersion of the nanoparticles. The highly negative surface of CMC-FeS confirmed that the attachment of CMC on FeS nanoparticles induced strong electrostatic repulsion, thereby preventing the particles from agglomeration. At the dosage of 10 mg/L as Fe, the dissolved Fe concentration of CMC-FeS was 0.20 mg/L, indicating that the nanoparticles remained essentially undissolved throughout the exposure period.

### Acute toxicity evaluation

The results showed that CMC-FeS were acutely toxic to zebrafish with a LC_50_ concentration of 21 mg/L as Fe upon the 96 h static exposure. Consequently, the nanoparticle dosage at the LC_25_ was used in the subsequent exposure experiments (LC_25_ is the highest exposure concentration possible to ensure adequate live organisms for downstream gene expression analysis^37^). The exposure to LC_25_ for 96 h resulted in no fish mortality, and no visible difference in behavior between control and treated group was observed. However, the exposure to 100 mg/L as Fe of the NPs (the maximum tested concentration) caused 100% mortality in 12 h. Moreover, aggressive behavior was observed within the 1 h of treatment showing a sign of toxicity stress. For instance, it was observed that the swimming speed and respiratory rate were increased, and fish were trying to jump out of the solution. The surface respiration took place and ultimately the fish lost their balance and presented jerky movements and sank on the bottom of the beaker before death. Extravasation of the blood was observed in the anterior ventral surface of the body, behind the head of fish. But, no such behavioral changes and extravasation of blood were observed at lower dosages of the CMC-FeS (5 or 10 mg/L as Fe) and the pigment of color was normal in all the fish.

Total iron concentrations in the fish carcasses after 96 h were determined to be 12.71 ± 1.63 mg/kg iron in the control group, and 28.16 ± 3.93 mg/kg for the CMC-FeS treated group, showing a 2.2 times Fe concentration increase after the exposure.

### Histological analysis of liver tissue

**Figure 2:**
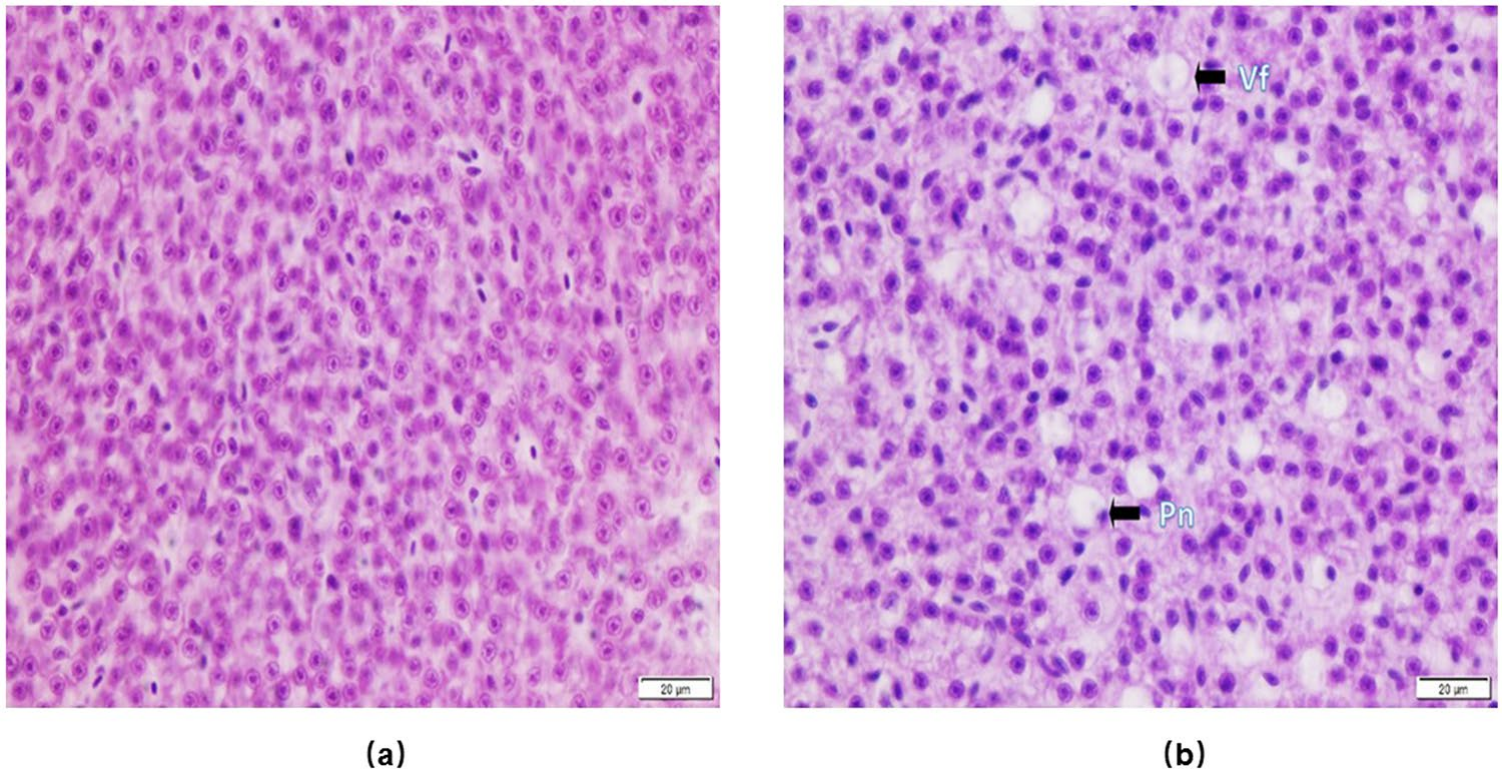
Histological analysis of the liver of Zebrafish in control (**a**) and upon CMC-FeS exposure (**b**). He liver of control zebrafish had normal hepatocytes, while the nanoparticle treated groups showed pyknotic nuclei (pn), and vacuole formation (vf). The scale bar represents 20 μm.

Histological alternation analysis of liver tissue in response to the CMC-FeS treatment were carried out via H&E staining to understand the possible penetration of the nanoparticles in liver. The control group showed normal hepatocytes (Fig. 2a), while the treated group showed cells with pyknotic nuclei and the presence of vacuolization, suggesting early stages of apoptosis (Fig. 2b) and clear signs of toxic effects.

### Transcriptomic analysis

The effect of CMC-FeS on zebrafish was further explored by screening the genes involved in toxicity and analyzing GO and KEGG pathways using the RNA-seq technology. The results revealed that the exposure and/or accumulation of CMC-FeS induced dramatic alterations in the global gene transcription profiles in the zebrafish livers.

The differences in the gene regulatory pathways were analyzed with the liver tissue. It produced approximately 263,707,236 total clean reads from the 39.6 Gbp clean sequence data for all the biological replicates. More than 88% of the clean reads had quality scores over the Q30 value, and over 83% of the total reads were uniquely mapped to the reference genome for the livers (Table 1).

**Table 1:** Summary of sequence data generated for zebrafish transcriptome and quality filtering.

Samples	Clean reads	Mapped reads	Mapped rate (%)	Q30 percentage (%)
Control-1	37,220,362	32,336,730	86.88	91.69
Control-2	41,194,526	34,871,813	84.65	96.16
Control-3	67,918,028	57,520,603	84.69	95.85
CMC-FeS NPs-1	40,250,516	34,530,413	85.79	88.49
CMC-FeS NPs-2	35,741,238	30,230,176	84.58	94.13
CMC-FeS NPs-3	41,382,566	34,632,427	83.69	94.68

**Figure 3:**
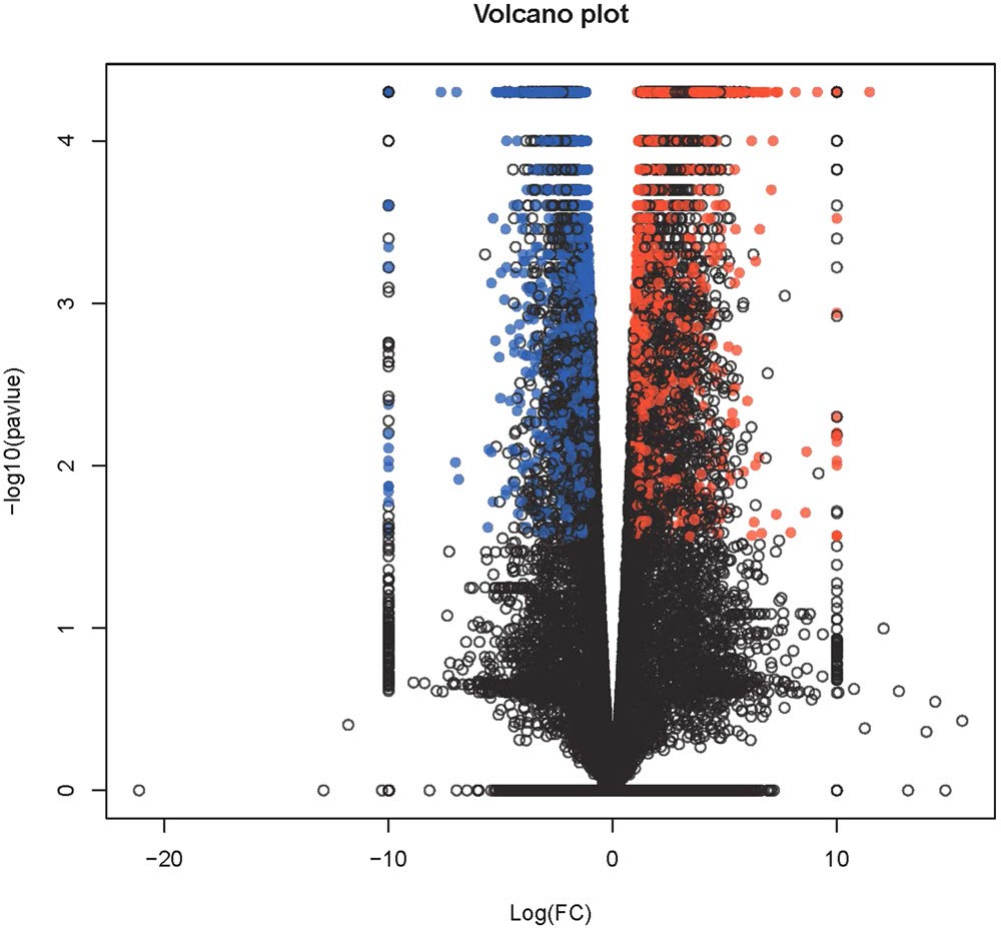
Volcano plot for the liver gene libraries of the CMC-FeS treated group and control group of zebrafish by showing variance in gene expression with respect to fold change (FC) and significance (P-value). Each dot represents an individual gene: Red dots represent the up-regulated DEGs (2593), Blue dots represent the down-regulated DEGs (3200), and black dots represent not differentially expressed genes (26472).

Relative to the control group, the volcano plots were constructed by integrating both the P value and fold change of each transcript (P-value < 0.05 and absolute log_2_ (fold change) >1), to show the general scattering of the transcripts and to filter the differentially expressed genes for the CMC-FeS treated group (Fig. 3). A large portion (55.2% or 3,200 genes) were down-regulated in the nanoparticle treated group, while 44.8% or 2,593 genes were up-regulated. Among the DEGs for treated samples, the observed striking differences in gene expression indicated a strong stress condition for zebrafish. Based on the FPKM value, 6 genes were expressed only in the treated fish, with two of them being up-regulated (*cmc4*, *pimr214*) and 4 down-regulated (*hist1h4l*, *hist1h4a*, *ighv5-1*, *dut*). These genes may play important roles in the physiological process and they are quite sensitive to the nanoparticles-induced stress.

**Figure 4:**
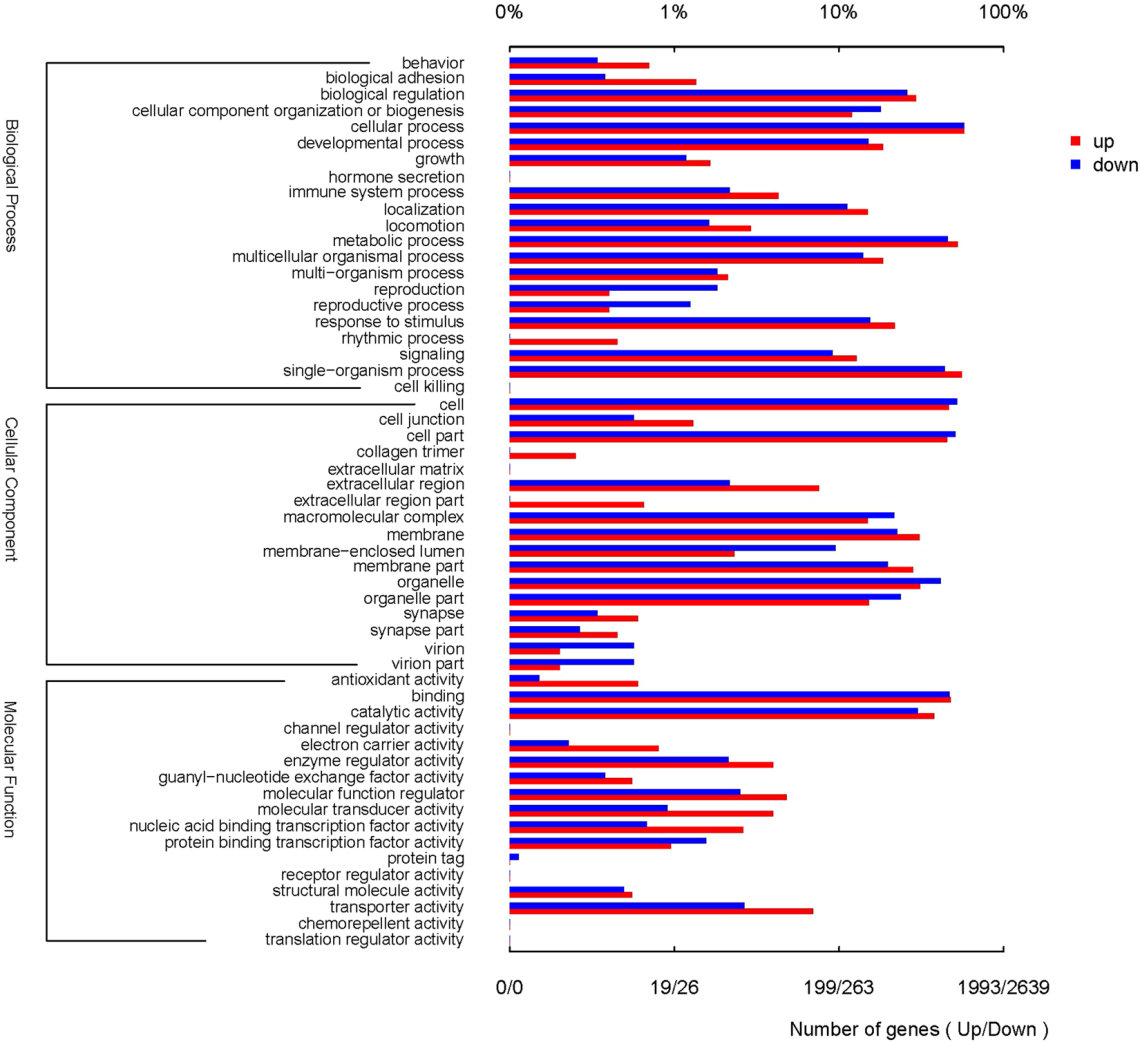
Gene ontology categories pattern of the differentially expressed genes in CMC-FeS treated group. Distribution of the GO categories was assigned into three categories: cellular components, molecular functions, and biological processes.

To identify the transcriptomic pathways affected by the exposure, the subset of genes that were significantly affected by the nanoparticle treatment were subjected to gene ontology (GO) analysis using the Goatools functional enrichment tool. The analysis was performed to identify GO pathways at cellular components, biological processes and molecular function categories. The basic GO unit is the GO term. Every GO term belongs to one of the particular category. GO terms with Bonferroni-corrected P-values < 0.05 were defined as being significantly enriched in DEGs. Figure 4 gives a histogram of the number and percentage of genes falling into the GO categories.

The majority of the responsive GO terms were found in the biological processes (1,392 GO terms, or 64.2%), followed by molecular function (510 GO terms, or 23.6%) and cellular components (265 GO terms, or 12.2%). In liver, the biological processes that were significantly enriched were mainly involved in metabolic process (GO:0008152), cellular process (GO:0009987), and response to stimulus (GO:0050896). The most DEGs-affected molecular functions were associated with catalytic activity (GO:0003824), transporter activity (GO:0005215), binding (GO:0005488), and molecular function regulator (GO:0098772). With regard to cellular components, cell part (GO:0044464), organelle (GO:0043226), macromolecular complex (GO:0032991) and organelle part (GO:0044422) were the largest DEGs categories.

To estimate the biological attributes of nanoparticles-specific genes, KEGG pathway analyses were carried out using DEGs excluding the genes shared with the control. A total of 46 significantly up-regulated KEGG pathways were observed in the livers of the nanoparticle treated group. Among them, ribosome, complement and coagulation cascades, glycine, serine and threonine metabolism, PPAR signaling pathway, and peroxisome were the five most significantly up-regulated pathways according to the P value (P < 0.05), while 37 KEGG pathways were down-regulated (P < 0.05), where cell cycle, DNA replication, pyrimidine metabolism, cell cycle-yeast and spliceosome were the most suppressed pathways. The pathway enrichment analysis based on loading stress-responsive genes identified significantly stress-related pathways. Table 2 lists the top ten up or down-regulated KEGG pathways based on the P-values.

**Table 2:** Top 10 differentially up or down-regulated KEGG pathways in CMC-FeS treated zebrafish compared with the control.

#	Pathways	Number of DEGs	P-Value	Pathway ID
**Up-regulated**				
1	Ribosome	76	1.38E-28	ko03010
2	Complement and coagulation cascades	35	3.18E-13	ko04610
3	Glycine, serine and threonine metabolism	22	2.42E-08	ko00260
4	PPAR signaling pathway	23	5.66E-07	ko03320
5	Peroxisome	27	2.00E-06	ko04146
6	Staphylococcus aureus infection	15	9.22E-06	ko05150
7	Tryptophan metabolism	18	1.14E-05	ko00380
8	Glyoxylate and dicarboxylate metabolism	13	6.84E-05	ko00630
9	Glycerolipid metabolism	18	7.59E-05	ko00561
10	Fat digestion and absorption	13	1.87E-04	ko04975
**Down-regulated**				
1	Cell cycle	48	3.05E-16	ko04110
2	DNA replication	23	2.34E-12	ko03030
3	Pyrimidine metabolism	35	6.35E-12	ko00240
4	Cell cycle - yeast	30	2.81E-11	ko04111
5	Spliceosome	36	5.47E-10	ko03040
6	RNA transport	37	2.41E-09	ko03013
7	RNA degradation	24	1.10E-07	ko03018
8	Meiosis - yeast	21	3.81E-07	ko04113
9	Purine metabolism	36	3.92E-07	ko00230
10	Ubiquitin mediated proteolysis	30	8.64E-07	ko04120

Several members of genes involved in responses to immune and inflammatory response, detoxification, oxidative stress, and DNA damage/repair were selected for RT-qPCR validation. As an internal gene, the beta-actin was used. Under the given experimental conditions, the internal gene was not affected by the exposure to the nanoparticles (data not shown). Generally, as shown in Fig. S1, the gene expression levels and trend of regulation (increase/decrease) detected with RNA-seq and qualification based on qPCR are in agreement with each other.

## Discussion

It is generally accepted that chemicals/toxicants first elicit biological changes at the molecular level. Toxicant-induced dysregulation of gene expression may subsequently lead to biological changes at the cellular level. The liver as the key innate immune organ for detoxification is very susceptible to damage. Typically, histological observation of key immune organ can reveal abnormal immune cells/tissues as well as *in vivo* molecular changes.

Our results indicated that the iron concentration of CMC-FeS affected zebrafish carcasses was 2.2 times higher than in the control. Given the rather negative surface potential of CMC-FeS, direct surface binding is not favored between the NPs and the tissue surfaces. This accumulation of the nanoparticles resulted in observable morphological alterations in the liver tissue at the tested exposure concentrations and duration. As seen in the present study, the exposure to CMC-FeS caused significant cytological damage to the hepatocytes. The presence of cells with pyknotic nuclei and the presence of vacuolization suggest early stages of apoptosis, thereby reducing the detoxification processes of zebrafish. This agrees with an earlier study where histopathological changes such as pyknosis occurred in zebrafish liver after exposure to AgNP due to oxidative stress and apoptosis^38^.

Histological evidence of the liver stress was further confirmed by exploring the DEGs upon the CMC-FeS exposure. Over expression of *flot2a* (5-fold), which is encoding flotillin 2, a protein previously proposed as involved in nanoparticles transport into cells, suggested the nanoparticles were taken up by endocytosis in zebrafish liver cells^39–41^. The same gene family member *flot1* was found over expressed in liver of fathead minnow when exposed to PVP-coated Ag nanoparticles but not to AgNO_3_^42^. The expression pattern of this gene may provide insight for the nanoparticle sorption and the cellular uptake mechanism of CMC-FeS.

When introduced to organisms, NPs would have elevated levels of the transcripts, which are known to be induced during oxidative stress and other stress conditions^43^. This study intended to address the acute toxicity of CMC-FeS on zebrafish. Therefore, the time resolved response of oxidative stress and other stress conditions, for instance, cell cycle damage, were confined within 96 h. Similar approach was employed by others^44–46^. Interestingly, our results indicated that CMC-FeS can cause up-regulation of some biomarker genes for inflammation and oxidative stress. The *cp* gene, coding for ceruloplasmin, is an inflammatory-linked gene that was expressed at a higher level, was found 18.5-fold more in the treated group compared with the control. Ceruloplasmin is an acute phase protein that is synthesized in liver during the acute phase of an inflammatory response and it also plays a key role in iron detoxification; *cp*, also considered as a metalloprotein gene, was found to perform the primary functions of all of the encoded proteins concerning metal ion binding and metal detoxification in birds^47^, as such, it is considered as an indicator of acute inflammation when birds are concerned^48^. Furthermore, *stat2*, a well-known essential and specific positive effector of type I interferons (IFN) signaling, was over expressed (12.6-fold) in liver; IFNs are multifunctional cytokines that regulate immune responses and cellular functions^49^, and activation of *stat2* can cause corresponding inflammation response. The third differentially expressed gene, *tsc22d3*, encoding a leucine zipper transcription factor, was found up-regulated. Several studies showed that *tsc22d3* is induced by glucocorticoids (GCs), and it plays a very important role as mediator in the anti-inflammatory and immunosuppressive action of GCs^50^. Regulation of *tsc22d3* gene was found in response to 5 nm AuNPs exposure (300 μM, 72 h) in Caco-2 cells showing inflammation stress conditions^51^. Moreover, multiple immune sensor genes, e.g. *crp*, *b2m* and *b2ml* were up-regulated; immune effector genes, e.g., complement components (such as *c3a.1*, *c3a.2*, *c8a*, *c9*) were identified over-expressed in the liver transcriptome when exposed to CMC-FeS. The up-regulation of these biomarker genes indicated the inflammation response and iron detoxification effect when adult zebrafish were exposed to CMC-FeS.

In addition, *pdia4*, the protein product of an endoplasmic reticulum (ER) chaperones, was found down-regulated (5-fold) in our study, showing the evidence for decreased ER function due to stress. A previous report indicated that ER stress in PC12 cells induced by over-expression of β-amyloid precursor protein was also accompanied by the up-regulation of *sgk1* expression^52^. This finding was consistent with our result that *sgk1* was over expressed (3.7-fold) in the CMC-FeS treated group. Similar finding was reported for zebrafish hepatic inflammation when exposed to Tris (1,3-dichloro2-propyl) Phosphate (TDCIPP)^53^. It was reported that ER stress can directly induce toll-like receptors (TLRs) and synergize with TLRs to cause inflammatory responses or/and related disease in liver^54^. Collectively, the up-regulation of inflammatory biomarker genes and induced ER stress strongly suggested that inflammatory response did occur in the zebrafish liver upon the CMC-FeS exposure.

Inflammation triggers an increased production of reactive oxygen species (ROS). If not effectively removed by antioxidant defenses, ROS can lead to oxidative stress and damage to cellular macromolecules such as nucleic acids, lipids and proteins^55^. Thus, inflammation and oxidative stress are tightly linked.

Oxidative stress has been proposed as a possible mechanism involved in the toxicity of nanoparticles^56^. In our study, the CMC-FeS exposure increased the expression of several oxidative stress related genes such as *sod3a* (19.8-fold) and *cat* (5.4-fold), suggesting the production of ROS. The *cat* gene induction was considered as an indicator of ROS production when zebrafish was exposed to uranium^57^. The up-regulation of gene expression for constituents of mitochondrial dysfunction pathway (genes including *mt-nd4*, *mt-nd5*, *mt-cyb*, *cox4l1*, *cox6b1*, *mt-co2*, and *mt-co3*) further suggested the production of ROS in the CMC-FeS treated group. The results agree with a previous study showing the alternation of these mitochondrial dysfunction pathway genes when zebrafish embryos were exposed to Ag nanoparticles, bulk Ag and Ag^+^ by transcriptome analysis^45^. Another stress indicator gene, cytochrome P450 1 A (*cyp1a*) was found up-regulated (12-fold) upon the CMC-FeS exposure, indicating detoxification of the stressor. Concurrently, *abcb4* (ATP-binding cassette, sub-family B (MDR/TAP), member 4) was significantly up-regulated in the treated group. The ATP-binding cassette (ABC) superfamily of genes encode membrane proteins that transport a diverse set of substrates across membranes. These genes play important roles in protecting organisms from xenobiotics^58^. It was reported that *abcb4* could affect bioavailability of chemicals to zebrafish embryos^58^ and adult tissues^59^. The ATP-binding cassette superfamily of genes was also found over-expressed in liver and gill of zebrafish when exposed to uranium^57^. Meanwhile, another gene family member, *abcb11b*, was induced in liver, highlighting the role of liver in the detoxification process.

Many studies have ascribed the toxicity of iron-based nanoparticles to ROS induced oxidative stress, which may cause DNA damage, tissue damage and cell death^60–62^. In this work, the DNA integrity may have been affected because *rad51b* was found down-regulated in liver, which can be attributed to the excessive production of ROS. *rad51b* is *rad51* paralog b. The *rad51* paralogs play important roles in the maintenance of genomic integrity through recombinational repair^63^. Similarly, the origin recognition complexes (*orc1*, *orc3* and *orc5*), which are essential proteins for DNA replication, were found down-regulated, indicating the negative effect on the cell cycle progression^64^. The significance of the very pronounced increase in *krt18* (12.5-fold), and hepatotoxicity biomarker gene^53^, further implied an apparent hepatoxicity.

The KEGG pathway analysis also provides evidence for toxic effects within the liver tissue. Several KEGG pathways were significantly altered, including those related with fish immunity such as complement and coagulation cascades, and genetic information processing such as ribosome, and cellular processes such as cell cycle.

The most significantly enriched KEGG pathway was ribosome. Some of the most pronounced changes were observed for the ribosomal proteins (e.g. *rpl10*, *rpl10a*, *rpl13*, *rpl14*, *rpl15*, *rpl18*, and *rpl18a*). Over expression of these genes was considered as an implication of modification of the primary metabolism, such as protein biosynthesis, and it is one of the major strategies that cells use to deal with stress. These results align well with previous literatures that reported disruption to protein biosynthesis pathway in a range of organisms following exposure to Ag nanoparticles and Ag^+ ^^45,65,66^. When Bluntsnout bream (*Megalobrama amblycephala*) was subjected to nitrite (15 and 30 mg/L), ribosome was found to be the most significantly enriched KEGG pathway^67^.

The complement and coagulation cascades pathway, related to the immune system response, was the second significantly up-regulated pathway that showed gene expression changes in the treated group. The activation of the complement cascade can be triggered by different signals involving various proteins. The protein C3 is always activated when this cascade is activated. Its activation represents a key event of the complement cascade, which will cause elimination of the nanoparticles that triggered the activation^68^. In this work, *c3* was found over expressed upon the CMC-FeS exposure. Moreover, the *f2* gene, which plays an important role in blood clotting, was dramatically up-regulated (14.4-fold), resulting in increased coagulation. The *f2* encodes a protein called prothrombin (also called coagulation factor II), which circulates in the bloodstream in an inactive form until an injury occurs that damages blood vessels. Prothrombin is converted to its active form, thrombin, when it is injured. Thrombin then converts fibrinogen into fibrin, the primary protein that makes up blood clots. Thrombin is also considered as a factor involved in cell growth and division, tissue repair, and the formation of new blood vessels^69^. The over expression of *c3* and *f2* indicated the immune system response upon the CMC-FeS exposure. This pathway was found significantly altered in rat during exposure to copper nanoparticles^70^, and it was also affected in bluntsnout bream upon nitrite exposure^67^.

The cell cycle pathway was the most significant down-regulated transcriptomic regulation KEGG pathway. The nanoparticles influenced the cell cycle pathway by down-regulating some of the key genes involved, such as cyclin A and cyclin B (*ccna1*, *ccna2*, *ccnb1*, *ccnb2*, and *ccnb3*) coding cyclins, and cyclin-dependent kinases (CDK) (*cdk1*, *cdk7*, *cdk8*, *cdk9*, and *cdk10*). The cylins function in the regulation of cell cycle progression and are associated with CDKs, which regulate cell growth, survival, differentiation and oncogenesis. Cell-cycle progression is well connected with the regulation of DNA damage repair^71^, and the down-regulation of cell cycle pathways indicates a possibility of cell death through apoptosis^72^. Other cell cycle regulators, origin recognition complexes (ORC) (*orc1*, *orc3*, and *orc5*) and mini-chromosome maintenance protein (MCM) complexes (*mcm10*, *mcm2* and *mcm5*), which are essential proteins for DNA replication, were all down-regulated, again implicating interference with cell cycle progression under the nanoparticle exposure. This is consistent with the previous study that the suppression of cell cycle regulators was accompanied by the down-regulation of a number of genes directly involved in the process of DNA replication (MCM10, ORC1) when tumor cells were subjected to fullerene for 48 h^64^.

## Conclusions

This is the first description of the relevance of major transcriptional changes associated with biological networks alternation upon exposure to engineered FeS NPs. This work provided both global and specific information on coordinated adaptive response to acute toxicity of CMC-FeS in the liver of zebrafish. Toxicity and transcriptome sequencing analyses demonstrated that the transcriptional activity associated with stress remained significant throughout the study. The findings were further supported by histological evidence, which may be used as a reference for phenotype-anchoring points for certain classes of genes in future studies. Since many differentially expressed genes are associated with immune and inflammatory response, detoxification, oxidative stress, and DNA damage/repair, our results indicated that exposure to CMC-FeS at LC_25_ for 96 h caused significant DNA and protein damage due to the nanoparticle induced oxidative stress. The oxidative stress caused major cellular and tissue injury, which was evident from the histological of NP-exposed liver and aligned well with changes in the altered expression of genes associated with hepatoxicity. The findings of this study provided insights into the genotoxicity and the toxicological mechanism caused by stabilized nanoparticles, which are useful for assessment of the potential toxicity of CMC-FeS and possibly other stabilized nanoparticles.

## Electronic supplementary material

Supplementary Information

## References

[1] Gong Y, Tang J, Zhao D (2016). Application of iron sulfide particles for groundwater and soil remediation: A review. Water Research.

[2] Jeong HY, Hayes KF (2007). Reductive dechlorination of tetrachloroethylene and trichloroethylene by mackinawite (FeS) in the presence of metals: reaction rates. Environmental Science & Technology.

[3] Skyllberg U, Drott A (2010). Competition between disordered iron sulfide and natural organic matter associated thiols for mercury (II)• An EXAFS study. Environmental Science & Technology.

[4] Mullet M, Boursiquot S, Ehrhardt J-J (2004). Removal of hexavalent chromium from solutions by mackinawite, tetragonal FeS. Colloids and Surfaces A: Physicochemical and Engineering Aspects.

[5] Han Y-S, Jeong HY, Demond AH, Hayes KF (2011). X-ray absorption and photoelectron spectroscopic study of the association of As (III) with nanoparticulate FeS and FeS-coated sand. Water Research.

[6] Han Y-S, Gallegos TJ, Demond AH, Hayes KF (2011). FeS-coated sand for removal of arsenic (III) under anaerobic conditions in permeable reactive barriers. Water Research.

[7] Hyun SP, Davis JA, Sun K, Hayes KF (2012). Uranium (VI) reduction by iron (II) monosulfide mackinawite. Environmental Science & Technology.

[8] Livens FR (2004). X-ray absorption spectroscopy studies of reactions of technetium, uranium and neptunium with mackinawite. Journal of Environmental Radioactivity.

[9] Butler EC, Hayes KF (1998). Effects of solution composition and pH on the reductive dechlorination of hexachloroethane by iron sulfide. Environmental Science & Technology.

[10] Oh S-Y, Kang S-G, Kim D-W, Chiu PC (2011). Degradation of 2, 4-dinitrotoluene by persulfate activated with iron sulfides. Chemical Engineering Journal.

[11] Watson, J., Ellwood, D., Pavoni, B., Lazzari, L. & Sperni, L. Degradation and removal of sediment PCBs using microbially generated iron sulfide. *Remediation and Beneficial Reuse of Contaminated Sediments*, 147–148 (2001).

[12] Gong Y, Liu Y, Xiong Z, Kaback D, Zhao D (2012). Immobilization of mercury in field soil and sediment using carboxymethyl cellulose stabilized iron sulfide nanoparticles. Nanotechnology.

[13] Gong Y, Liu Y, Xiong Z, Zhao D (2014). Immobilization of mercury by carboxymethyl cellulose stabilized iron sulfide nanoparticles: reaction mechanisms and effects of stabilizer and water chemistry. Environmental Science & Technology.

[14] Chen KL, Elimelech M (2007). Influence of humic acid on the aggregation kinetics of fullerene (C 60) nanoparticles in monovalent and divalent electrolyte solutions. Journal of Colloid and Interface Science.

[15] Domingos RF, Tufenkji N, Wilkinson KJ (2009). Aggregation of titanium dioxide nanoparticles: role of a fulvic acid. Environmental Science & Technology.

[16] Johnson RL, Johnson GOB, Nurmi JT, Tratnyek PG (2009). Natural organic matter enhanced mobility of nano zerovalent iron. Environmental Science & Technology.

[17] He F, Zhao D (2007). Manipulating the size and dispersibility of zerovalent iron nanoparticles by use of carboxymethyl cellulose stabilizers. Environmental Science & Technology.

[18] Liang Q, Zhao D (2014). Immobilization of arsenate in a sandy loam soil using starch-stabilized magnetite nanoparticles. Journal of Hazardous Materials.

[19] An B, Liang Q, Zhao D (2011). Removal of arsenic (V) from spent ion exchange brine using a new class of starch-bridged magnetite nanoparticles. Water Research.

[20] Scown T, Van Aerle R, Tyler C (2010). Review: do engineered nanoparticles pose a significant threat to the aquatic environment?. Critical Reviews in Toxicology.

[21] Hatton B, Rickard D (2008). Nucleic acids bind to nanoparticulate iron (II) monosulphide in aqueous solutions. Origins of Life and Evolution of Biospheres.

[22] Rickard D (2011). FeS-induced radical formation and its effect on plasmid DNA. Aquatic Geochemistry.

[23] Higgins, M. R. Environmental assessment of *in situ* groundwater remediation with reduced iron reactive media, *The University of Michigan*, (2011).

[24] Pastor J (2017). Effects of sulfate and sulfide on the life cycle of Zizania palustris in hydroponic and mesocosm experiments. Ecological Applications.

[25] Hill AJ, Teraoka H, Heideman W, Peterson RE (2005). Zebrafish as a model vertebrate for investigating chemical toxicity. Toxicological Sciences.

[26] Bar‐Ilan O, Albrecht RM, Fako VE, Furgeson DY (2009). Toxicity assessments of multisized gold and silver nanoparticles in zebrafish embryos. Small.

[27] OECD Guideline 203: Guidelines for testing of chemicals: fish, aquatic toxicity test, Organization for Economic Co-operation and Development, pp. 1–9 (1992).

[28] Chen H (2012). Sequence mining and transcript profiling to explore differentially expressed genes associated with lipid biosynthesis during soybean seed development. BMC Plant Biology.

[29] Sheehan, D. C. & Hrapchak, B. B. *Theory and practice of histotechnology*. (Cv Mosby, 1980).

[30] Patel, R. K. & Jain, M. NGS QC Toolkit: A Toolkit for Quality Control of Next Generation Sequencing Data. *Plos One* **7**, ARTN e30619 10.1371/journal.pone.0030619 (2012). 10.1371/journal.pone.0030619 PMC3270013 22312429

[31] Chen S (2014). Whole-genome sequence of a flatfish provides insights into ZW sex chromosome evolution and adaptation to a benthic lifestyle. Nature Genetics.

[32] Kim, D. & Salzberg, S. L. TopHat-Fusion: an algorithm for discovery of novel fusion transcripts. *Genome biolog*y **12**, Artn R72 10.1186/Gb-2011-12-8-R72 (2011). 10.1186/gb-2011-12-8-r72 PMC3245612 21835007

[33] Trapnell C (2013). Differential analysis of gene regulation at transcript resolution with RNA-seq. Nature Biotechnology.

[34] Xie C (2011). KOBAS 2.0: a web server for annotation and identification of enriched pathways and diseases. Nucleic Acids Res.

[35] Audic S, Claverie J-M (1997). The significance of digital gene expression profiles. Genome Research.

[36] Benjamini, Y. & Yekutieli, D. The control of the false discovery rate in multiple testing under dependency. *Annals of Statistics*, 1165–1188 (2001).

[37] Poynton HC (2010). Differential gene expression in Daphnia magna suggests distinct modes of action and bioavailability for ZnO nanoparticles and Zn ions. Environmental Science & Technology.

[38] Choi JE (2010). Induction of oxidative stress and apoptosis by silver nanoparticles in the liver of adult zebrafish. Aquatic Toxicology.

[39] Poynton HC (2012). Toxicogenomic responses of nanotoxicity in Daphnia magna exposed to silver nitrate and coated silver nanoparticles. Environmental Science & Technology.

[40] Freese C (2013). Uptake of poly (2-hydroxypropylmethacrylamide)-coated gold nanoparticles in microvascular endothelial cells and transport across the blood–brain barrier. Biomaterials Science.

[41] Kasper J (2013). Interactions of silica nanoparticles with lung epithelial cells and the association to flotillins. Archives of Toxicology.

[42] Garcia-Reyero NL (2014). Differential effects and potential adverse outcomes of ionic silver and silver nanoparticles *in vivo* and *in vitro*. Environmental Science & Technology.

[43] Simon DF (2013). Transcriptome sequencing (RNA-seq) analysis of the effects of metal nanoparticle exposure on the transcriptome of Chlamydomonas reinhardtii. Applied and Environmental Microbiology.

[44] Hou J, Zhou Y, Wang C, Li S, Wang X (2017). Toxic Effects and Molecular Mechanism of Different Types of Silver Nanoparticles to the Aquatic Crustacean Daphnia magna. Environmental Science & Technology.

[45] van Aerle R (2013). Molecular mechanisms of toxicity of silver nanoparticles in zebrafish embryos. Environmental Science & Technology.

[46] Wang Z-J (2016). Transcriptome profiling analysis of rare minnow (Gobiocypris rarus) gills after waterborne cadmium exposure. Comparative Biochemistry and Physiology Part D: Genomics and Proteomics.

[47] Watson, H., Videvall, E., Andersson, M. N. & Isaksson, C. Transcriptome analysis of a wild bird reveals physiological responses to the urban environment. *Scientific Reports* **7** (2017). 10.1038/srep44180 PMC5349542 28290496

[48] Chamanza R (1999). Serum amyloid a and transferrin in chicken. A preliminary investigation of using acute‐phase variables to assess diseases in chickens. Veterinary Quarterly.

[49] Li Z (2010). Administration of recombinant IFN1 protects zebrafish (Danio rerio) from ISKNV infection. Fish & Shellfish Immunology.

[50] Ayroldi E, Riccardi C (2009). Glucocorticoid-induced leucine zipper (GILZ): a new important mediator of glucocorticoid action. The FASEB Journal.

[51] Bajak E (2015). Changes in Caco-2 cells transcriptome profiles upon exposure to gold nanoparticles. Toxicology Letters.

[52] Copanaki E (2007). The amyloid precursor protein potentiates CHOP induction and cell death in response to ER Ca 2+depletion. Biochimica et Biophysica Acta (BBA)-Molecular Cell Research.

[53] Liu, C. *et al*. Acute exposure to tris (1, 3-dichloro-2-propyl) phosphate (TDCIPP) causes hepatic inflammation and leads to hepatotoxicity in zebrafish. *Scientific Reports* **6** (2016). 10.1038/srep19045 PMC4705469 26743178

[54] Lawless M, Greene C (2012). Toll-like receptor signalling in liver disease: ER stress the missing link?. Cytokine.

[55] Halliwell, B. & Gutteridge, J. M. *Free radicals in biology and medicine*. (Oxford University Press, USA, 2015).

[56] Nel A, Xia T, Mädler L, Li N (2006). Toxic potential of materials at the nanolevel. Science.

[57] Lerebours A (2009). Comparative analysis of gene expression in brain, liver, skeletal muscles, and gills of zebrafish (Danio rerio) exposed to environmentally relevant waterborne uranium concentrations. Environmental Toxicology and Chemistry.

[58] Dean M, Annilo T (2005). Evolution of the ATP-binding cassette (ABC) transporter superfamily in vertebrates. Annual Reiew of Genomics and Human Genetics.

[59] Lu X (2015). Zebrafish Abcb4 is a potential efflux transporter of microcystin-LR. Comparative Biochemistry and Physiology Part C: Toxicology & Pharmacology.

[60] Keenan CR, Goth-Goldstein R, Lucas D, Sedlak DL (2009). Oxidative stress induced by zero-valent iron nanoparticles and Fe (II) in human bronchial epithelial cells. Environmental Science & Technology.

[61] Li H (2009). Effects of waterborne nano-iron on medaka (Oryzias latipes): antioxidant enzymatic activity, lipid peroxidation and histopathology. Ecotoxicology and Environmental Safety.

[62] Wu H, Yin J-J, Wamer WG, Zeng M, Lo YM (2014). Reactive oxygen species-related activities of nano-iron metal and nano-iron oxides. Journal of Food and Drug Analysis.

[63] Yokoyama H (2004). Preferential binding to branched DNA strands and strand‐annealing activity of the human Rad51B, Rad51C, Rad51D and Xrcc2 protein complex. Nucleic Acids Research.

[64] Lucafò M (2013). Profiling the molecular mechanism of fullerene cytotoxicity on tumor cells by RNA-seq. Toxicology.

[65] Nair PMG, Choi J (2011). Characterization of a ribosomal protein L15 cDNA from Chironomus riparius (Diptera; Chironomidae): transcriptional regulation by cadmium and silver nanoparticles. Comparative Biochemistry and Physiology Part B: Biochemistry and Molecular Biology.

[66] Powers CM, Badireddy AR, Ryde IT, Seidler FJ, Slotkin TA (2011). Silver nanoparticles compromise neurodevelopment in PC12 cells: critical contributions of silver ion, particle size, coating, and composition. Environmental Health Perspectives.

[67] Sun S, Ge X, Xuan F, Zhu J, Yu N (2014). Nitrite-induced hepatotoxicity in bluntsnout bream (Megalobrama amblycephala): the mechanistic insight from transcriptome to physiology analysis. Environmental Toxicology and Pharmacology.

[68] Vauthier C, Persson B, Lindner P, Cabane B (2011). Protein adsorption and complement activation for di-block copolymer nanoparticles. Biomaterials.

[69] Markiewski MM, Nilsson B, Ekdahl KN, Mollnes TE, Lambris JD (2007). Complement and coagulation: strangers or partners in crime?. Trends in Immunology.

[70] Liao M, Liu H (2012). Gene expression profiling of nephrotoxicity from copper nanoparticles in rats after repeated oral administration. Environmental Toxicology and Pharmacology.

[71] Panigrahi S, Mai S (2005). Telomeres, genomic instability, DNA repair and Breast Cancer. Current Medicinal Chemistry-Anti-Inflammatory & Anti-Allergy Agents.

[72] Frohlich E (2013). Cellular targets and mechanisms in the cytotoxic action of non-biodegradable engineered nanoparticles. Current Drug Metabolism.

