# Supplementary material for: Toxicity and Transcriptome Sequencing (RNA-seq) Analyses of Adult Zebrafish in Response to Exposure Carboxymethyl Cellulose Stabilized Iron Sulfide Nanoparticles
Source: Sci Rep. 2018 May 24;8:8083. doi: 10.1038/s41598-018-26499-x (PMC5967324; doi:10.1038/s41598-018-26499-x)
Supplement: Supplementary file 1 — Supplementary Information [file 41598_2018_26499_MOESM1_ESM.docx]

**Supporting Information**

**Toxicity and Transcriptome Sequencing (RNA-seq) Analyses of Adult Zebrafish in Response to Exposure Carboxymethyl Cellulose Stabilized Iron Sulfide Nanoparticles**

**Min Zheng^1,2^, Jianguo Lu^2^, Dongye Zhao^1,3*^**

*^1^ Environmental Engineering Program, Department of Civil Engineering, Auburn University, Auburn, AL, 36849, USA*

*^2^School of Marine Sciences, Sun Yat-sen University, Guangdong, 510275, China*

*^3^Beijing University of Civil Engineering and Architecture, Beijing 100044, PR China*

**^*^**C*orresponding author:* [zhaodon@auburn.edu](mailto:zhaodon@auburn.edu)

**Table of contents**

**Supplementary Tables**

Table S1

Table S2

**Supplementary Figure**

Fig. S1

**Table S1.** Main water quality parameters for local tap water

| **Parameters** | **pH** | **Conductivity**  (µS/cm) | **Ca (mg/L)** | **Mg (mg/L)** | **Al**  **(mg/L)** | **Fe (mg/L)** | **Cl^-^ (mg/L)** | **SO_4_^2-^ (mg/L)** | **Cu**  **(μg/L)** | **Hardness (mg/L)** |
| --- | --- | --- | --- | --- | --- | --- | --- | --- | --- | --- |
| Values | 7.12 | 338 | 11.970 | 5.892 | 0.015 | 0.027 | 5.95 | 5.35 | 7.29 | 54.153 |

**Table S2.** Sequences of primers for selected genes.

| **Gene Name** | **Forward primer** | **Reverse primer** |
| --- | --- | --- |
| ***flot2a*** | CATTCATACGAGGCGAGCAG | CCTTATTCGAGGACGACGGA |
| ***cp*** | GGACTGGGAAATGAGGTGGA | GACAGTGCAGTAGCCATGTG |
| ***stat2*** | GTCGGAAATCTCGGCTATGC | CTTCTGGAGCTGGAACATGC |
| ***tsc22d3*** | GCCTTTCCAAGTCAAGCCAA | GCTCTGTTACAGGTCCGTCT |
| ***sgk1*** | GAAAGGGTAGCTTCGGCAAG | GAGTAATGCAGGCCCACAAG |
| ***sod3a*** | AGTAAACGCAGTGGGAATGC | CAGATGAGGCTTGGTGATGC |
| ***cyp1a*** | TGGAGCTAATTGGCACTGGA | TAGGCGCATGAGCAGATACA |
| ***abcb4*** | TGGCCTGACGTTCTCTTTCT | CTCTCCAACTGCCATTGCTC |
| ***krt18*** | GAGTGCAAGTGGTAGCACAG | CCAGACGGTCGTTCAAGTTC |
| ***pdia4*** | AGGTCCAGACCCTGAAACAG | ATACGCTGCATCTTCATCGC |
| ***rad51b*** | TAAGACAGCAGTCCTGCACA | GGGAACAGGAGTCATGGGAA |
| ***orc1*** | GGCAGCTCTTTCAGGTGATG | CACTGCCTTTCTGCTGGTTT |

**Figure S1.** Validation of liver tissue transcriptome results by qRT-PCR using twelve selected differentially expressed genes in CMC-FeS treated zebrafish. The qRT-PCR fold changes are relative to the control samples and normalized by changes in beta-actin values. The averages of three relative quantities of biological replications were used in a two-tailed Student’s t test with a 95% confidence level (P<0.05) to determine the gene expression significance.
